# Glymphatic system dysfunction in temporal lobe epilepsy with hippocampal sclerosis: MRI-based evaluation of circulatory markers and disease progression

**DOI:** 10.3389/fnins.2026.1741257

**Published:** 2026-02-19

**Authors:** Yingpeng Kuang, Dingwen Hu, Hui Chu, Rongyu Zhang, Ziyu Diao, Kun Wang, Xiaomei Yue, Yuna Chen, Shijun Qiu, Jie An

**Affiliations:** 1 The First Clinical Medical College, Guangzhou University of Chinese Medicine, Guangzhou, China; 2 Department of Radiology, The First Affiliated Hospital of Guangzhou University of Chinese Medicine, Guangzhou, China; 3 State Key Laboratory of Traditional Chinese Medicine Syndrome, Guangzhou, China

**Keywords:** disease duration, glymphatic system, hippocampal sclerosis, MRI, neuroimaging, temporal lobe epilepsy

## Abstract

**Objectives:**

In this study, several MRI-derived glymphatic markers were utilized to evaluate alterations in glymphatic system (GS) function in patients with temporal lobe epilepsy and hippocampal sclerosis (TLE-HS).

**Methods:**

we assessed 160 participants, including 80 patients with TLE-HS and 80 healthy controls (HCs). Glymphatic circulation was evaluated using MRI-based markers, including choroid plexus volume (CPV), perivascular space (PVS) volume, the fraction of white matter free water (FW-WM), and diffusion tensor imaging along the perivascular space (DTI-ALPS) index. Group differences were evaluated using two-sample tests, and associations were assessed using partial Spearman’s rank correlations (ρ).

**Results:**

In patients with TLE-HS, the DTI-ALPS index (*t* = −2.65, *P* = 0.001), CPV/TIV (*t* = −2.65, *P* = 0.001), and PVS score [χ^2^,test χ^2^ (3, *N* = 160) = 15.21, *p* = 0.0016] were significantly lower than in HCs, whereas the FW-WM (*t* = 5.70, *P* < 0.001) was significantly higher. Additionally, longer disease duration was significantly correlated with a decrease in DTI-ALPS (ρ = −0.375, *P* = 0.001), an increase in FW-WM (ρ = 0.316, *P* = 0.001), and an enlargement of CPV (ρ = 0.378, *P* = 0.001). Furthermore, a reduction in hippocampal volume (HPV) was closely correlative with a decrease in DTI-ALPS (ρ = 0.226, *P* = 0.048) and an enlargement of CPV (ρ = −0.345, *P* = 0.002).

**Conclusion:**

This study offers substantiated evidence of GS function in patients with TLE-HS using multiple MRI-derived indices of GS function. Moreover, longer disease duration was significantly associated with lower DTI-ALPS indices, higher FW-WM levels, and enlarged CPV, while reduced HPV was closely linked to decreased DTI-ALPS and increased CPV. These findings indicate that MRI-derived GS indices may serve as potential imaging biomarkers of disease chronicity in TLE-HS.

## 1 Introduction

The glymphatic system (GS), consists of perivascular channels that enable exchange between cerebrospinal fluid (CSF) and interstitial fluid (ISF). First characterized in rodent models, this pathway supports convection-driven elimination of interstitial solutes and metabolic by-products from brain parenchyma (Iliff et al., 2012). Disruption of this pathway is associated with neurological disorders, and its disturbance is increasingly quantifiable through surrogate metrics derived from magnetic resonance imaging (MRI) (Rasmussen et al., 2018; Ringstad et al., 2018).

Temporal lobe epilepsy with hippocampal sclerosis (TLE-HS) is considered to be a prototypical form of focal epilepsy, marked by chronic neuroinflammation, gliopathy, and progressive network remodeling (Blümcke et al., 2013). GS dysfunction may alter perivascular water exchange and CSF dynamics around the medial temporal lobe and thereby contribute to its pathophysiology (Hlauschek et al., 2024); however, direct *in vivo* evidence remains limited. Recent studies suggest that the enlarged perivascular spaces (ePVS) in TLE-HS may localize to temporal-lobe structures and relate to clinical course, supporting a perivascular contribution to disease progression (Feldman et al., 2018; Cho et al., 2025).

Several MRI-derived indices provide complementary windows for assessing glymphatic function. The diffusion tensor imaging along the perivascular space (DTI-ALPS) index leverages anisotropic water diffusion orthogonal to principal fiber tracts to approximate perivascular flow along the medullary veins and has shown sensitivity to flow alterations across multiple disorders (Taoka et al., 2017; Taoka and Naganawa, 2020). The free water fraction in white matter (FW-WM) can reflect expansion of the extracellular water compartment due to edema or neuroinflammation—processes that arise when exchange is impaired (Pasternak et al., 2009). Finally, choroid plexus volume (CPV), a putative marker of CSF-production capacity and immune activity, has emerged as a disease-relevant measure under neuroinflammatory conditions ^(^ Xu et al., 2024; De Meo et al., 2025).

It should be noted, however, that these single metrics can be confounded by underlying pathology and imaging factors—PVS is influenced by age, small vessel disease, and image resolution (Barisano et al., 2022); FW-WM may be affected by edema or inflammation (Mayer et al., 2022); DTI-ALPS is sensitive to motion and sequence parameters (Haller et al., 2024); and CPV or permeability is not specific to GS function (Choi et al., 2022). Therefore, a multimodal or multi-index approach may better capture GS function by integrating complementary dimensions such as structural burden of the perivascular pathway, diffusional flux, and CSF–subarachnoid space exchange (Naganawa et al., 2024). Recent evidence from multicenter cohorts and longitudinal designs shows that these MRI-based measures are reproducible under varied acquisition protocols and are increasingly employed as validated biomarkers of GS function in neurodegenerative and metabolic disorders (Li H. et al., 2024). Nevertheless, to date, no prior work has comprehensively evaluated GS function in patients with TLE-HS using this combination of metrics.

Building on these advances, we investigated cerebral edema–related alterations in TLE-HS using a multimodal MRI panel comprising the DTI-ALPS index, FW-WM fraction, the burden of ePVS, and CPV. Importantly, the primary contribution of the present work is to evaluate these glymphatic-related markers simultaneously within the same cohort and to characterize their concordance and complementarity, rather than relying on a single surrogate measure. We further examined whether these indices were associated with disease duration and hippocampal volume (HPV), given prior reports linking disease duration, hippocampal atrophy, and putative glymphatic impairment. We prespecified that, relative to healthy controls (HCs), patients would exhibit altered glymphatic markers, and that longer disease duration and smaller HPV would be associated with a more “dysfunctional” glymphatic profile.

## 2 Materials and methods

### 2.1 Participants

Patients with TLE-HS were retrospectively enrolled according to the following inclusion criteria: (1) seizure type and clinical manifestations consistent with the diagnostic and classification criteria established by the International League Against Epilepsy (ILAE) in 2017 (Scheffer et al., 2017); (2) interictal or ictal electroencephalography (EEG) showing epileptiform discharges originating from the temporal lobe; (4) adults aged 18–50 years; and (5) MRI with fluid-attenuated inversion recovery (FLAIR) sequences revealing hippocampal hyperintensity and volume loss. The following exclusion criteria were employed: (1) space-occupying intracranial lesions (e.g., tumors), history of head trauma, or psychiatric disorders; (2) bilateral HS; and (3) Individuals with neurodegenerative conditions. Hippocampal sclerosis laterality (left vs. right) was determined on routine clinical MRI by experienced neuroradiologists. Only unilateral HS cases were included in the present cohort (left, *n* = 45; right, *n* = 35).

The present study was conducted on a cohort of 80 subjects diagnosed with TLE-HS, as determined by the enrollment committee from the Department of Encephalopathy, the First Affiliated Hospital of Guangzhou University of Chinese Medicine, and 80 HCs were recruited from the local community. Screening MRI was unremarkable in all controls, and none had a previous neurologic or psychiatric illnesses. Clinical characteristics of all TLE-HS patients were extracted from medical records, and all patients were receiving antiepileptic drugs (AEDs) therapy at the time of inclusion. Ethical approval was obtained from the Ethics Committee of the First Affiliated Hospital of Guangzhou University of Chinese Medicine (No. 82471942). All procedures adhered to the Declaration of Helsinki, and each participant received study information and signed informed consent prior to enrollment.

### 2.2 MRI data acquisition

The MRI data acquisition process was conducted on a 3-T Siemens MAGNETOM Prisma scanner, equipped with a 64-channel head coil. T2-weighted and FLAIR images were acquired to screen for structural abnormalities. For structural neuroimaging, three-dimensional T1-weighted (3D-T1w) sequence was obtained (TR/TE/TI = 2,530/2.98/1,100 ms, flip angle = 7°; FOV = 256 × 256 mm^2^; 192 sagittal slices; slice thickness = 1.0 mm; voxel size = 1.0 × 1.0 × 1.0 mm^3^). All participants were instructed to remain motionless, keep their eyelids closed, and maintain wakefulness for the duration of the acquisition. In addition, Diffusion tensor imaging (DTI) scans were acquired with single-shot echo-planar imaging (SS-EPI) and parallel imaging. We collected 25 non-collinear diffusion directions at b = 1,000 s/mm^2^ plus one b0 image (b = 0 s/mm^2^). Sequence parameters were: TR/TE = 12,000/75.5 ms, flip angle = 90°, number of excitations = 1, FOV = 24 × 24 cm^2^, matrix = 128 × 128, slice thickness = 3 mm, and no inter-slice gap. Total scan time was 5 min 36 s.

### 2.3 MRI data preprocessing

The 3D-T1W images were processed using a toolbox known as Computational Anatomy Toolbox (CAT12^1^), which was implemented using SPM12^2^. The segmentation of brain tissue was conducted, subsequently followed by spatial registration and normalization utilizing the DARTEL algorithm. On this basis, the total intracranial volume (TIV) was subsequently calculated. In order to preserve the local gray matter volume information of each voxel, the segmented images were modulated using the Jacobian determinants obtained during the spatial normalization process. Hippocampal volumes (HPV) in the left and right hemispheres were extracted and summed based on the LPBA40 brain atlas (Shattuck et al., 2008). To minimize interindividual differences in brain anatomy, HPV was normalized by TIV, yielding the standardized total hippocampal volume (HPV/TIV).

**FIGURE 1:**
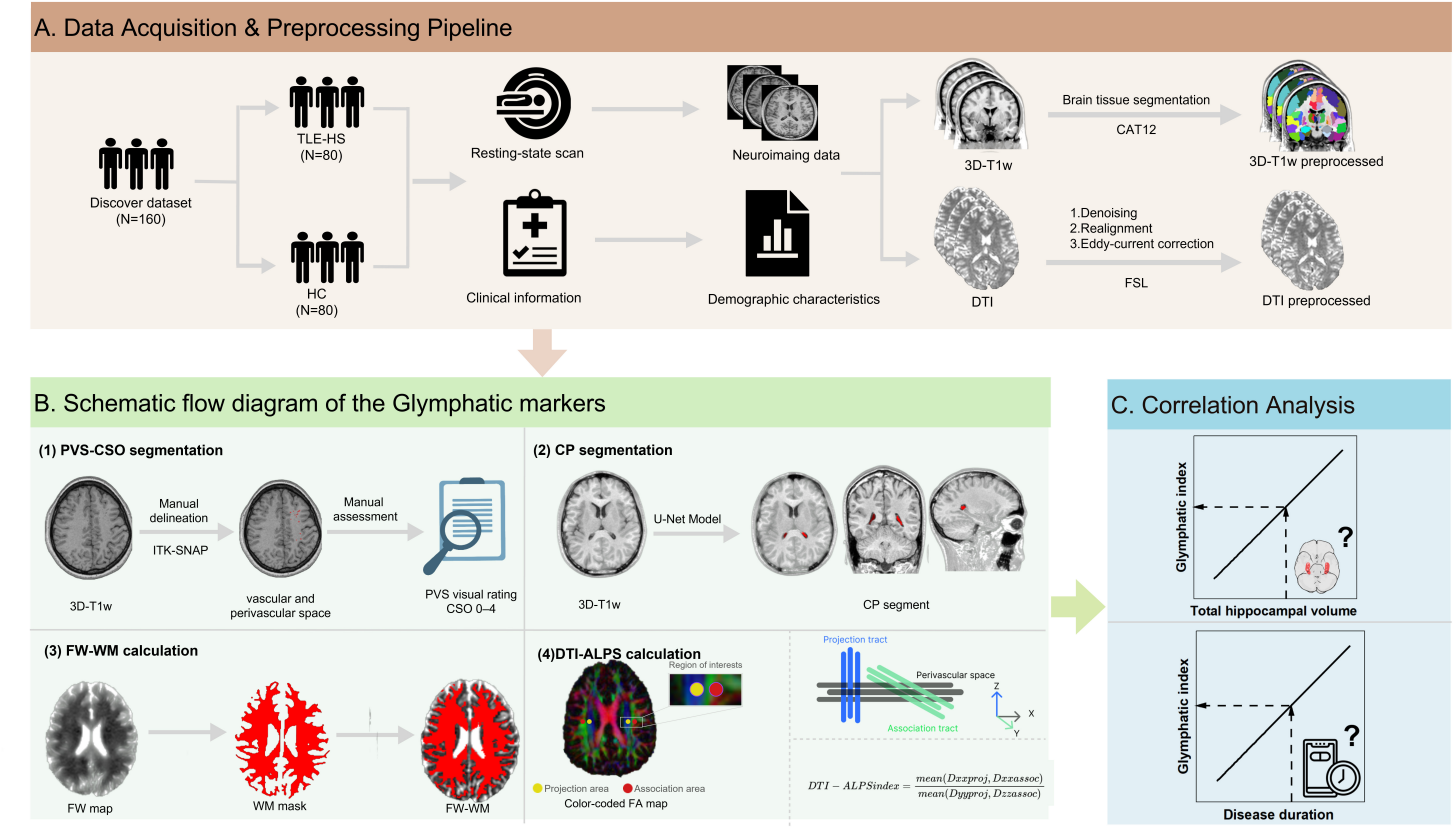
Study design and analytic workflow. **(A)** Data acquisition and preprocessing. The cohort included patients with unilateral temporal lobe epilepsy with hippocampal sclerosis (TLE-HS; *n* = 80) and healthy controls (HCs; *n* = 80). Structural three-dimensional T1-weighted (3D-T1w) images were segmented with CAT12. Diffusion tensor imaging (DTI) data underwent denoising, realignment and eddy-current correction in FSL. Clinical and demographic variables were recorded. **(B)** Derivation of glymphatic indices. Perivascular spaces in the centrum semiovale (PVS-CSO) were visually rated (Potter 0–4) after manual delineation on 3D-T1w images. Choroid plexus (CP) was segmented on 3D-T1w images using a U-Net model and normalized by total intracranial volume (CPV/TIV). Free water fraction in white matter (FW-WM) was obtained from free-water maps within a white-matter mask. The DTI-ALPS index was computed from 5-mm spherical ROIs placed at the level of the lateral ventricle body in projection and association fiber regions on color-coded FA maps, using the formula ALPS = mean (Dxx_proj, Dxx_assoc)/mean (Dyy_proj, Dzz_assoc). **(C)** Association analyses. Relationships between glymphatic indices and total hippocampal volume and disease duration were assessed by correlation analysis. DTI-ALPS, the diffusion tensor imaging along the perivascular space; FSL, FMRIB Software Library; ROI, region of interest.

In addition, individual white matter masks were constructed for each participant as follows. Firstly, 3D-T1w images were preprocessed using FreeSurfer 7.3.2^3^ to generate subject-specific white matter masks. The DTI data underwent preprocessing using the FMRIB Software Library 6.0 (FSL^4^). DTI was acquired with a single phase-encoding direction and only one non-diffusion-weighted (b0) volume; no reverse phase-encoding b0 images were collected. Therefore, susceptibility-induced EPI distortions were not explicitly corrected using TOPUP. Firstly, the fslmaths command was used to generate maps of mean, standard deviation, and signal-to-noise ratio (SNR) to comprehensively inspect potential artifacts within each image. Subsequently, the FMRIB Diffusion Toolbox (FDT) was utilized in the generation of fractional anisotropy (FA) maps. The specific preprocessing steps were as follows: (1) eddy-current and motion-induced distortions were corrected with eddy_correct; (2) subject-specific brain masks were generated from the non-diffusion-weighted (b0) volume using the Brain Extraction Tool (BET) with a fractional intensity threshold of 0.25; and (3) diffusion tensors were fitted with DTIFIT, thereby allowing the derivation of FA and mean diffusivity (MD) maps for each participant (Figure 1A).

### 2.4 Computation of MRI-derived markers of GS function

#### 2.4.1 Choroid plexus volume derivation

In this study, we applied a three-dimensional, fully convolutional network with a U-Net design to automatically delineate the choroid plexus (CP) within the lateral ventricles from 3D-T1w images (Eisma et al., 2024). The processing procedure was as follows: first, the 3D-T1w images were input into the model for segmentation inference; second, the segmentation outputs were spatially inverse-transformed to map the results back to each subject’s native imaging space; Third, the calculation of CPV was performed based on the generated segmentation masks. To enhance the accuracy and reliability of segmentation results, two experienced radiologists manually reviewed and corrected the automatically segmented images. To minimize the influence of interindividual anatomical variability, the CPV was normalized to the TIV, yielding the standardized choroid plexus volume (CPV/TIV) (Figure 1B, panel 2).

#### 2.4.2 Semi-quantitative evaluation of perivascular spaces

Perivascular space were visually evaluated on 3D-T1w images for all participants using the semi-quantitative visual rating scale proposed by Potter et al. (2015). Specifically, the hemisphere with the higher PVS burden was selected for scoring. The quantity of PVS present within the centrum semiovale (CSO) was counted and classified into five grades: grade 0, no visible PVS; grade 1, 1–10 visible PVS; grade 2, 11–20 visible PVS; grade 3, 21–40 visible PVS; and grade 4, more than 40 visible PVS. All images were independently assessed by two experienced clinical neuroradiologists. If discrepancies arose between the two experienced radiologists, the final rating was determined by consensus after joint discussion (Figure 1B, panel 1).

#### 2.4.3 FW-WM calculation

The FW-WM estimation was executed using scripts that had been made available by the MarkVCID consortium^5^. This method is based on a bi-tensor model, which represents each voxel as a mixture of an isotropic free-water compartment and an anisotropic tissue tensor (Zhou et al., 2023). The processing pipeline included the following steps: first, extracellular free water was quantified to obtain an isotropic free-water compartment and its fractional volume; second, DTI modeling was repeated after removing the influence of free water to extract anisotropic tissue components, thereby generating free-water–corrected DTI metrics, the resulting FW maps represented the voxel-wise fractional volume of free water, ranging from 0 to 1; Third, the white matter mask was registered to the FW maps using the corresponding b0 images, and the mean free water value within the FW-WM was extracted (Figure 1B, panel 3). No additional thresholding on FW values was applied beyond the anatomical white matter masking step.

#### 2.4.4 DTI-ALPS calculation

The DTI data were subjected to a series of preprocessing steps using FSL software (see text footnote 4), after which diffusion tensor fitting was executed with the DTIFIT tool to ascertain diffusivities along the three principal axes (Dxx, Dyy, and Dzz). Based on previously established methods, the relevant diffusion components were then extracted from the fitted tensor parameters to calculate the DTI-ALPS index (Taoka et al., 2017). Specifically, the x-axis diffusivity was used as an indicator of diffusion along perivascular spaces as well as within projection fibers (Dxx_proj) and association fibers (Dxx_assoc). Meanwhile, the y-axis diffusivity of projection fibers (Dyy_proj) and the z-axis diffusivity of association fibers (Dzz_assoc) were extracted as normalization references. According to the JHU-ICBM-DTI-81 white matter atlas, spherical regions of interest (ROIs; 5 mm in diameter) were manually placed in the projection and association fiber regions at the level of the body of the lateral ventricles in both hemispheres (Figure 1B, panel 4; Mori et al., 2005). Finally, the DTI-ALPS index was calculated as Taoka et al. (2017):


$$D\,T\,I-A\,L\,P\,S\,i\,n\,d\,e\,x=\frac{m\,e\,a\,n\,\left(D\,x\,x\,p\,r\,o\,j,D\,x\,x\,a\,s\,s\,o\,c\right)}{m\,e\,a\,n\,\left(D\,y\,y\,p\,r\,o\,j,D\,z\,z\,a\,s\,s\,o\,c\right)}$$

The DTI-ALPS index was computed separately for the left and right hemispheres. For laterality-aligned analyses, hemisphere-specific ALPS values were relabeled as ipsilateral and contralateral relative to the HS side; unless otherwise specified, the bilateral mean DTI-ALPS value was used for group-level comparisons.

### 2.5 Statistics analysis

Analyses were carried out with SPSS v26.0 (IBM Corp., Armonk, NY, USA). For continuous variables, the analytic method was determined by distribution: data approximating normality were compared with independent-samples *t* tests, whereas non-normal data underwent analysis using the Mann-Whitney U test. For categorical variables, binary outcomes were evaluated with the chi-square test, and ordinal outcomes were modeled with ordinal logistic regression. Quantitative variables that adhere to a normal distribution are reported as the mean value ± the standard deviation (M ± SD). The non-normal variables are to be summarized using the median and interquartile range [M (Q1, Q3)]. Qualitative variables are presented as counts (%). Statistical significance was set at α = 0.05 (two-tailed).

Age, sex, years of education, and estimated TIV (eTIV) were included as covariates in general linear models (GLMs) to compare differences in normalized HPV/TIV and MRI-derived GS indices between the TLE-HS group and the HC group. Within the TLE-HS group, partial Spearman correlation analyses controlling for age, sex, eTIV, and education were performed to assess associations between MRI-derived GS indices and both HPV/TIV and clinical parameters (Figure 1C). For all analyses, the statistical significance was defined as α = 0.05. For laterality analyses within the TLE-HS group, ipsilateral and contralateral ALPS indices were compared using paired Wilcoxon signed-rank tests. Associations between ipsilateral ALPS and ipsilateral hippocampal volume, as well as disease duration, were assessed using covariate-adjusted partial Spearman correlations via a residualization approach controlling for age, sex, education, and TIV (two-tailed α = 0.05). To assess the similarity/covariance among the four MRI-derived glymphatic-related indices, we constructed a pairwise correlation matrix within the TLE-HS group. For each index, residuals were obtained after regressing out age, sex, TIV, and years of education (consistent with the partial correlation analyses above), and Spearman’s rank correlations (ρ) were computed between residualized indices. P values for the six pairwise correlations were adjusted using the Benjamini–Hochberg false discovery rate (FDR) procedure.

## 3 Results

### 3.1 Demographic characteristics

A comparison of the demographic and clinical characteristics of the TLE-HS group and the HC group can be found in Table 1. Compared with the HC group, the TLE-HS group had significantly fewer years of education (*P* < 0.001). There were no significant differences in age or sex between the two groups.

**TABLE 1:** Between-group differences for demographic and clinical characteristics, as well as magnetic resonance imaging (MRI) indexes of the glymphatic function measurements.

Characteristics	TLE-HS (*N* = 80)	HCs (*N* = 80)	*P*-value
**HS laterality, *n* (%)**			
Left	45 (56.2%)	–	–
Right	35 (43.8%)	–	–
sex, *n* (%)			0.341
Male	46 (57.5%)	40 (50.0%)	–
Female	34 (42.5%)	40 (50.0%)	–
Age (y), mean ± SD	27.57 ± 8.54	27.70 ± 6.65	0.919
Education (y), mean ± SD	10.23 ± 2.43	14.26 ± 2.83	<0.001
eTIV, mean ± SD	1398.41 ± 148.47	1451.30 ± 143.74	0.023
Duration (y), mean ± SD	10.51 ± 7.48	–	–
Onset age (y), mean ± SD	17.04 ± 8.76	–	–
PVS-CSO grade, *n* (%)			0.006
Grade-1	35 (43.8%)	59 (73.8%)	–
Grade-2	36 (45.0%)	17 (21.2%)	–
Grade-3	8 (10.0%)	3 (3.8%)	–
Grade-4	1 (1.2%)	1 (1.2%)	–
DTI-ALPS, mean ± SD	1.41 ± 0.12	1.47 ± 0.16	0.009
FW-WM, mean ± SD	0.20 ± 0.02	0.19 ± 0.01	<0.001
CPV/TIV, mean ± SD	0.15 ± 0.04	0.12 ± 0.03	<0.001
HPV/TIV, mean ± SD	0.27 ± 0.03	0.29 ± 0.02	<0.001

TLE-HS, temporal lobe epilepsy with hippocampal sclerosis; HCs, healthy controls; eTIV, estimated total intracranial volume; DTI-ALPS, the diffusion tensor imaging along the perivascular space; FW-WM, free water fraction in white matter; HPV/TIV, bilateral hippocampal volume/TIV × 100; CPV/TIV, choroid plexus volume/TIV × 100; PVS-CSO grade, semiquantitative white-matter perivascular space rating in the centrum semiovale (grades 1–4; higher grade indicates greater burden).

### 3.2 Alterations in MRI-based GS indexes in TLE-HS

**FIGURE 2:**
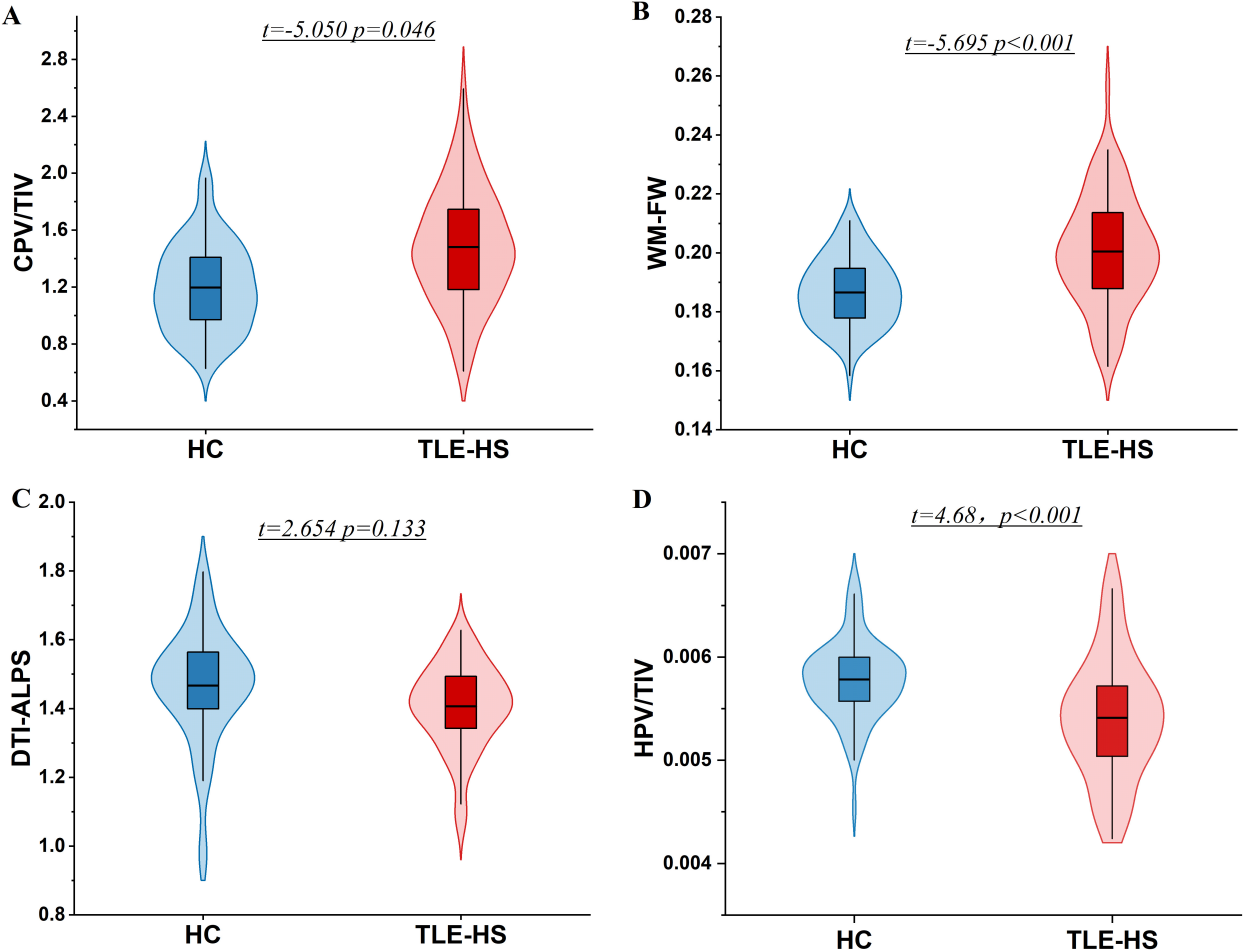
Group differences in MRI-derived GS indexes between TLE-HS and HCs. **(A)** Total CPV/TIV was higher in TLE-HS than in HCs. **(B)** Free water fraction in white matte (FW-WM) was higher in TLE-HS. **(C)** Mean DTI-ALPS was lower in TLE-HS. **(D)** HPV/TIV was lower in TLE-HS than in HCs. CPV, choroid plexus volume; FW-WM, free water fraction in white matter; DTI-ALPS, diffusion tensor image analysis along the perivascular space; HC, healthy control; TIV, total intracranial volume; TLE-HS, temporal lobe epilepsy with hippocampal sclerosis; HPV, hippocampal volume.

Following adjustment for variables including sex, age, and years of education, the TLE-HS group exhibited a reduced TIV in comparison with the HC group (*P* = 0.023), Therefore, eTIV was included as a covariate in all subsequent GLM and partial correlation analyses involving MRI-derived markers. In contrast, the normalized HPV/TIV was significantly lower in the TLE group than in the HC group (*t* = 4.68, *P* < 0.001) (Figure 2D).

In comparison with the HC group, the TLE-HS group exhibited significantly higher total CPV/TIV (*t* = −2.65, *P* = 0.001) (Figure 2A), and FW-WM (*t* = 5.70, *P* < 0.001) (Figure 2B). In contrast, the TLE group showed significantly lower mean DTI-ALPS indices (*t* = −2.65, *P* = 0.001) (Figure 2C). Moreover, the distribution of PVS-CSO Potter grades differed significantly between groups (χ^2^ test, χ^2^ (3, *N* = 160) = 15.21, *p* = 0.0016; Supplementary Figure 1), with higher PVS-CSO grades observed in the TLE-HS group.

### 3.3 Associations between the GS indexes and HPV/TIV in TLE-HS

**FIGURE 3:**
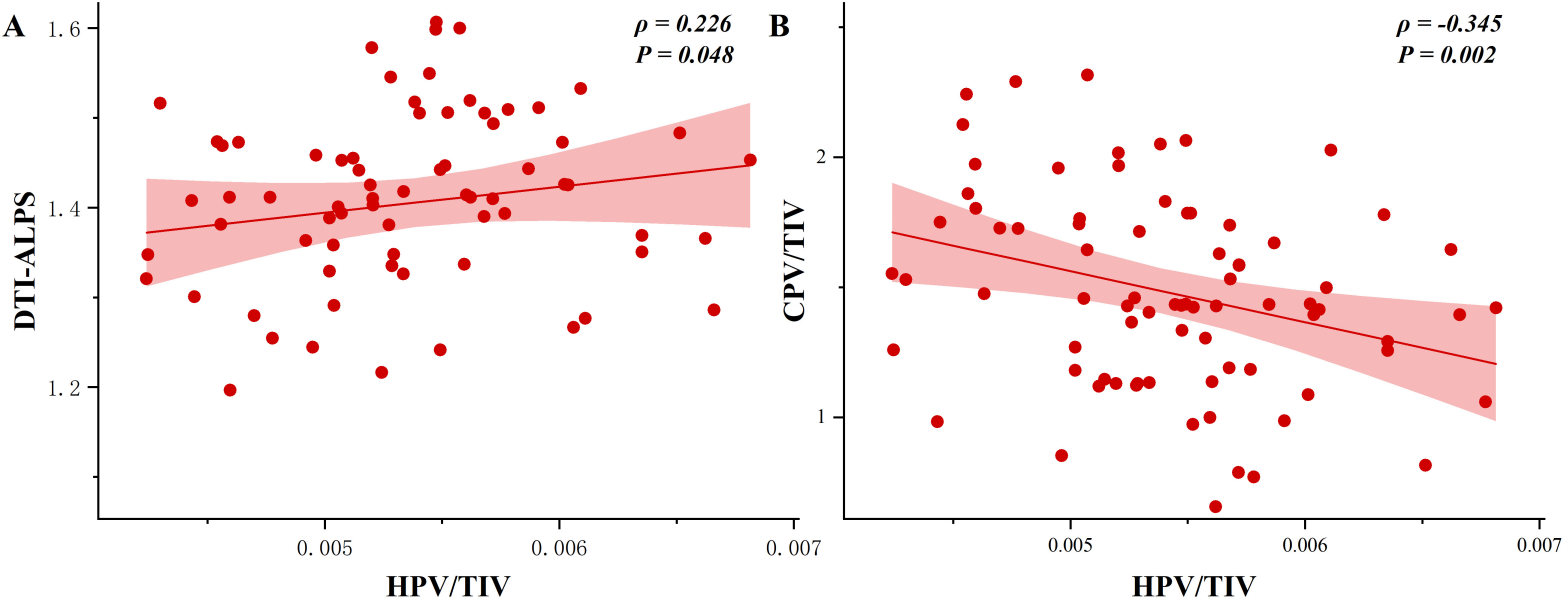
Associations between HPV/TIV and GS indexes in TLE-HS. **(A)** Scatterplot of DTI-ALPS versus HPV/TIV showing a positive association (ρ = 0.226, *P* = 0.048). **(B)** Scatterplot of total CPV/TIV versus HPV/TIV showing a negative association (ρ = –0.345, *P* = 0.002). DTI-ALPS, diffusion tensor image analysis along the perivascular space; CPV, choroid plexus volume; HPV, hippocampal volume; TIV, total intracranial volume; TLE-HS, temporal lobe epilepsy with hippocampal sclerosis.

Following adjustment for age, sex, years of education, and eTIV, partial Spearman correlation analyses were conducted. This analysis revealed a significant association between higher HPV/TIV and higher mean DTI-ALPS values within the TLE-HS group (ρ = 0.226, *P* = 0.048) (Figure 3A). In contrast, higher total HPV/TIV was significantly correlated with lower total CPV/TIV (ρ = −0.345, *P* = 0.002) (Figure 3B).

### 3.4 Associations between the GS indexes and disease duration in TLE-HS

**FIGURE 4:**
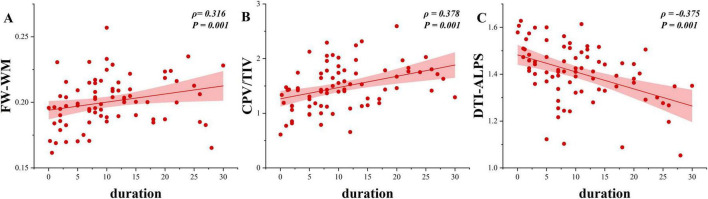
Associations between disease duration and MRI-derived GS indexes in TLE-HS. **(A)** Longer disease duration was associated with higher total CPV/TIV (ρ = 0.378, *P* = 0.001). **(B)** Longer duration was associated with higher FW-WM (ρ = 0.316, *P* = 0.001). **(C)** Longer duration was associated with lower mean DTI-ALPS (ρ = –0.375, *P* = 0.001). CPV, choroid plexus volume; FW-WM, free water fraction in white matter; DTI-ALPS, diffusion tensor image analysis along the perivascular space; HPV, hippocampal volume; TIV, total intracranial volume; TLE-HS, temporal lobe epilepsy with hippocampal sclerosis.

Furthermore, a longer disease duration was significantly associated with higher total CPV/TIV (ρ = 0.378, *P* = 0.001) (Figure 4B), and FW-WM (ρ = 0.316, *P* = 0.001) (Figure 4A). Conversely, longer disease duration was significantly correlated with lower mean DTI-ALPS (ρ = −0.375, *P* = 0.001) (Figure 4C). However, no significant correlations were found between the PVS-CSO Potter score and either disease duration or HPV/TIV (*P* = 0.372).

### 3.5 Interrelationships among MRI-derived glymphatic indices

**FIGURE 5:**
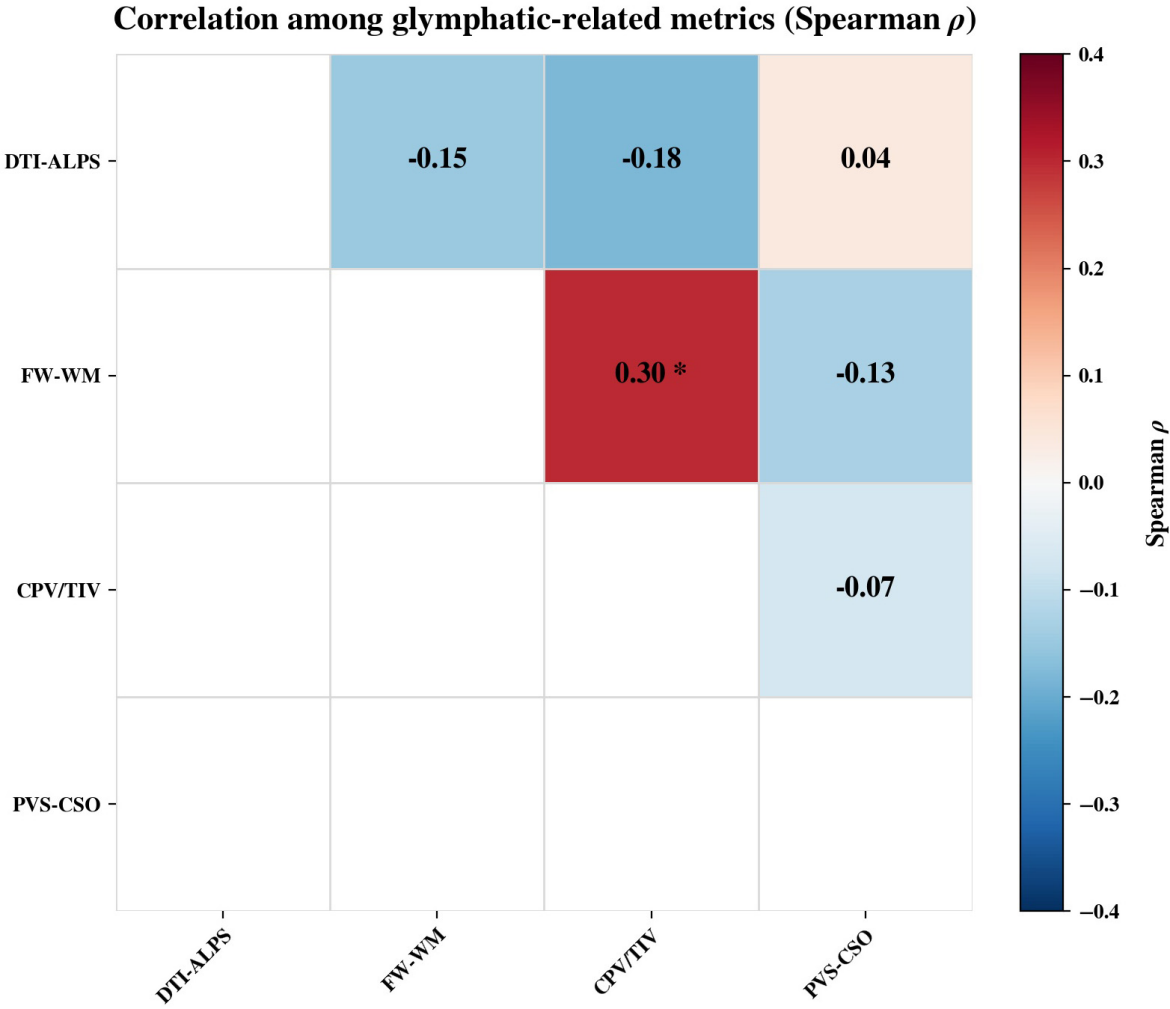
Correlation matrix of MRI-derived glymphatic-related indices in TLE-HS. Upper-triangular heatmap shows pairwise partial Spearman correlations (ρ) among DTI-ALPS, FW-WM, CPV/TIV, and PVS-CSO after adjustment for age, sex, and years of education. *Indicates significance after Benjamini–Hochberg FDR correction (q < 0.05).

To examine covariance across the multi-metric panel, we computed a partial Spearman correlation matrix among residualized indices (adjusting for age, sex, education and eTIV) within the TLE-HS group (Figure 5). FW-WM was modestly positively correlated with CPV/TIV (ρ = 0.30, FDR q < 0.05), whereas correlations among other pairs were weak (DTI-ALPS vs FW-WM: ρ = −0.15; DTI-ALPS vs. CPV/TIV: ρ = −0.18; DTI-ALPS vs. PVS-CSO: ρ = 0.04; FW-WM vs. PVS-CSO: ρ = −0.13; CPV/TIV vs. PVS-CSO: ρ = −0.07; all FDR q > 0.05).

### 3.6 Laterality-aligned analyses

Within the TLE-HS group, the DTI-ALPS index did not differ between the ipsilateral and contralateral hemispheres (paired Wilcoxon signed-rank test: W = 1,560, Z = −0.285, *P* = 0.776). After adjustment for age, sex, education, and eTIV, ipsilateral DTI-ALPS was positively associated with ipsilateral HPV (partial Spearman ρ = 0.31983, *P* = 0.00383; *n* = 80), whereas no association was observed with disease duration (partial Spearman ρ = −0.17689, *P* = 0.1165; *n* = 80).

## 4 Discussion

This study integrated multiple MRI-derived indices to evaluate GS-related alterations in TLE-HS and to examine their associations with HPV and disease duration. Given that group differences in individual markers have been reported previously, the primary contribution of this work lies in the simultaneous evaluation of a multi-metric panel and the interpretation of the findings as a coherent profile. Across the four indices, TLE-HS showed a consistent pattern characterized by lower DTI-ALPS together with higher FW-WM, larger CPV/TIV, and a greater PVS-CSO burden. Within the patient group, disease duration tracked three measures (lower DTI-ALPS, higher FW-WM, and higher CPV/TIV), whereas PVS-CSO showed no detectable association with duration. These results support a pattern-level view in which complementary MRI proxies jointly inform GS-related alterations, rather than relying on any single surrogate measure in isolation.

Contemporary models propose that CSF—largely produced by the choroid plexus—enters periarterial spaces and exchanges with ISF through convective (advection-dominated) transport coupled with diffusion; fluid then exits along perivenous pathways and cranial/spinal routes (Iliff et al., 2012; MacAulay et al., 2022). Increasing evidence suggests a close relationship between GS function and the onset and persistence of TLE (Lee et al., 2022; Sun Q. et al., 2024; Xie et al., 2024). On the one hand, recurrent seizures are associated with neuroinflammation and redox imbalance, as well as blood–brain barrier (BBB) disruption and alterations in the perivascular microenvironment (Vezzani et al., 2011; Pearson-Smith and Patel, 2017). These processes are linked to altered astrocytic polarity and redistribution of aquaporin-4 (AQP4), together with a marked reduction in the efficiency of CSF–ISF exchange. An increase in BBB permeability has been reported to coincide with the extravasation of blood-borne proteins and inflammatory mediators, and may be associated with augmented gliotic responses, homeostatic imbalance, and reduced cerebral glucose metabolism (Ivens et al., 2007). On the other hand, Microvascular remodeling or alterations in the architecture of PVS in patients with chronic epilepsy have been reported to be associated with changes in CSF dynamics and with a greater waste-clearance burden (van Lanen et al., 2021; Liu et al., 2024). When clearance capacity is impaired, these molecules may accumulate in vulnerable regions, such accumulation has been associated with glial and ionic dysregulation, aberrant plasticity, and network reorganization, potentially reflecting a lowered seizure threshold or heightened network susceptibility (Cho et al., 2025). This accumulation can lead to glial and ionic imbalance, abnormal plasticity, and network reorganization, which lowers the seizure threshold or increases network susceptibility (Iliff et al., 2012; Steinhäuser et al., 2012). A study by Xie et al. (2024) indicated a reduction in DTI-ALPS in TLE-HS, consistent with alterations in perivascular diffusion metrics.

However, DTI-ALPS is an indirect structural marker based on the directional diffusion ratio and does not directly measure CSF flow or metabolic clearance (Haller et al., 2024). Postmortem studies have shown that high DTI-ALPS can occur even in the absence of circulation (Li et al., 2025), suggesting that it is more sensitive to white matter orientation rather than “glymphatic flow.” The method’s developers have also explicitly stated that DTI-ALPS should not be equated with GS function (Taoka et al., 2024). Therefore, a multi-marker approach (e.g., PVS, FW-WM, CPV, and DTI-ALPS) should be used to enhance interpretability and robustness (Naganawa et al., 2024). Based on this, accumulating evidence indicates that DTI-ALPS alone does not provide a comprehensive characterization of the GS multidimensional features (Taoka et al., 2024). Different MRI-derived metrics provide complementary information: PVS primarily reflects the structural load and morphology of the perivascular spaces, the FW-WM indicates the extracellular free water component and is subject to influence by factors such as inflammation, edema, and microstructural changes, CPV characterizes the volume of the choroid plexus, the primary source of CSF, while DTI-ALPS indirectly reflects the directional diffusion of water molecules under specific regional geometric constraints (Barisano et al., 2022; Lee et al., 2022; Drenthen et al., 2024; Sun S. et al., 2024).

We found that the CPV was larger in patients with TLE-HS. Prior work in TLE (Wang et al., 2024) showed associations of CP enlargement and lower DTI-ALPS with poorer semantic fluency. That study further indicated that DTI-ALPS served as a mediator between CP enlargement and cognitive impairment. Given that the CP serves both the blood–CSF barrier and a regulator of CSF secretion, its inflammation and barrier or secretory dysregulation may perturb CSF dynamics and relate to GS function (Solár et al., 2020). Prior work has shown associations between CP alterations and impaired GS function (Li et al., 2023). However, CP enlargement is not invariably linearly related to clearance efficiency; its variation is modulated by factors such as age, vascular status, immune activation, and CSF production rate (Eisma et al., 2024; Li C. et al., 2024). Accordingly, CPV is best interpreted as one complementary marker within the multi-metric panel.

We found that the PVS-CSO score was significantly elevated in TLE-HS. In a study by Hlauschek et al. (2024), it was reported that the PVS burden in adult epilepsy patients increased, and that ePVS count and volume were correlated with the seizure duration. The potential mechanism for this enlargement may involve endothelial overactivation during seizures, leading to increased production of amyloid-β and tau proteins, which causes PVS obstruction. This results in the accumulation of neurotoxic waste within the PVS, which blocks the PVS and impedes lymphatic clearance, thereby worsening GS function (Perosa et al., 2022). However, analyses of Human Connectome Project data indicate that white-matter ePVS volume increases approximately linearly across the lifespan, whereas ePVS counts begin to decline from midlife onward. This suggests that ePVS presence is not only related to pathological states but also influenced by physiological factors such as age (Lynch et al., 2023). In our study, we reduced the potential influence of age by selecting age-matched TLE-HS patients and controls, ensuring consistency in the age distribution between the TLE-HS patients and the control group, thereby minimizing age-related confounding in PVS-CSO scores and GS function.

FW-WM was indication of the fraction of water molecules that are unrestricted or non-directional in their movement and is commonly regarded as an indirect imaging marker of interstitial fluid content (Pasternak et al., 2009). In the present study, FW-WM was significantly elevated in TLE-HS, which may indicate increased freely diffusible water in white matter and, indirectly, suggest potential ISF retention or stagnation. Consistent with our findings, previous studies by Sharma et al. (2023) also reported increased free water in epileptogenic brain regions in TLE. One possible mechanism is that CSF penetrates the brain parenchyma along periarterial spaces and exchanges with ISF via AQP4, thereby driving metabolic waste and ISF toward perivenous pathways for clearance back into the CSF circulation (Xie et al., 2024). Seizure activity may be associated with AQP4 dysfunction and glymphatic impairment, and educed GS–mediated clearance has been hypothesized to occur concomitantly with impaired ISF drainage and an increased FW-WM fraction (Nowicka, 2023). However, it is important to emphasize that FW-WM alone cannot be directly attributed to GS function. Elevated FW-WM may also result from edema, inflammatory infiltration, demyelination, BBB leakage, or gliosis, making it difficult to distinguish GS impairment from other pathological processes (Kern et al., 2023; Drenthen et al., 2024). Therefore, Given its non-specificity, FW-WM should be interpreted alongside the other MRI proxies in this panel when inferring GS-related alterations.

From a laterality perspective, we observed no detectable difference between ipsilateral and contralateral DTI-ALPS indices, suggesting that DTI-ALPS alterations in TLE-HS may not be strictly lesion-confined. Nonetheless, ipsilateral DTI-ALPS remained positively associated with ipsilateral HPV after covariate adjustment, supporting a link between local structural injury and this diffusion-based proxy. In contrast, the lack of association with disease duration indicates that duration alone may not adequately capture the cumulative pathological burden relevant to GS-related changes.

Taken together, our results support GS-related alterations in TLE-HS across a multi-metric MRI panel. Notably, the inter-metric correlation matrix suggests limited redundancy among markers: only FW-WM and CPV/TIV showed a modest shared variance, while other pairs were weakly correlated. This pattern indicates that the four indices likely capture partially distinct components of GS-related dysfunction (e.g., perivascular diffusional flux, extracellular water burden, CSF-CP involvement, and structural PVS burden), reinforcing the value of simultaneous multi-metric assessment for a more comprehensive characterization in TLE-HS.

In our cohort, total HPV correlated positively with the DTI-ALPS index, whereas total HPV correlated inversely with CPV/TIV. Taken together, these results indicate a statistical association between hippocampal structural measures and GS function. Despite the unresolved structural underpinnings of DTI-ALPS, our observations align with those of Roura et al. (2025), who reported a positive association between DTI-ALPS values and HPV in patients diagnosed as having isolated rapid-eye-movement sleep behavior disorder (iRBD). These convergent results are consistent with the proposed link between GS function and hippocampal structure (Roura et al., 2025). Such findings are in line with the proposed link among glymphatic activity, disease duration in TLE, and secondary functional and structural brain changes. Nevertheless, this remains a working hypothesis that warrants confirmation in future longitudinal studies examining the relationship between DTI-ALPS and other markers of GS function as well as brain structural and functional metrics. Similarly, Liu et al. (2025) reported an inverse association between CPV and the volumes of the hippocampus and amygdala in patients with systemic lupus erythematosus (SLE), consistent with our findings. The hippocampus, as a key component of the GS, is particularly susceptible to neuroinflammatory pathology. The CP, on the other hand, has been proposed as a neurogenic interface that participates in immune surveillance of the GS and modulates hippocampal neurogenesis (Planques et al., 2019). Notably, the hippocampus is critical for higher cognitive functions, including episodic memory and learning, and its structural integrity is closely associated with cognitive impairment in both neurodegenerative and epilepsy-related disorders (Tsalouchidou et al., 2023). The GS, which facilitates metabolic waste clearance and maintains synaptic homeostasis, has recently been suggested to influence cognitive performance (Nedergaard and Goldman, 2020). Therefore, a potential “structure–function–clearance” interaction model may exist among hippocampal integrity, cognitive capacity, and glymphatic flux. Although cognitive testing was not included in the present study, future longitudinal and mediation analyses integrating hippocampal morphology, glymphatic indices, and cognitive phenotypes may help elucidate the interplay between brain structural remodeling and clearance pathway dysfunction in TLE.

In addition, epileptic discharges and the persistence of seizures may further exacerbate CSF–ISF circulation dysfunction (Han et al., 2024). In our study, a longer disease duration was associated with diminished DTI-ALPS indices and elevated FW-WM and CPV/TIV, which supports this notion. The present findings align with those documented by Xie et al. (2024), Zhang et al. (2023), both of whom demonstrated reduced DTI-ALPS values in TLE. Furthermore, a negative correlation was noted between DTI-ALPS values and the disease duration. Similarly, Tsuda et al. (2018) reported widespread white matter integrity abnormalities in TLE that were correlated with the duration of epilepsy. It is noteworthy that evidence linking CPV to TLE disease duration remains scarce. However, in inflammatory disorders such as multiple sclerosis, CP enlargement has been shown to correlate with disease activity and progression (Klistorner et al., 2022). Experimental models indicate that AQP4 deficiency impairs glymphatic flow, resulting in reduced clearance of metabolic waste and heightened susceptibility to seizures (Sun Q. et al., 2024). Moreover, recent neuroimaging advances using fMRI and PET have revealed impaired glymphatic clearance in patients with epilepsy, characterized by reduced CSF flux through PVS and greater retention of neurotoxic proteins (Elabasy et al., 2023; Fonseca et al., 2024). Concordantly, both human and animal studies have shown BBB disruption, perivascular inflammation, and microhemorrhages in epilepsy (Jeon et al., 2013; Greene et al., 2022). Therefore, the chronicity of epilepsy may be associated with persistent abnormalities in GS-related indices.

Several limitations merit consideration. Firstly, the study design is characterized as a single-center, cross-sectional investigation, with the sample size being relatively modest. Consequently, the necessity for larger multicenter cohorts with longitudinal follow-up has been identified to confirm these findings and permit causal inference. Second, antiepileptic medications (ASMs) use was not systematically recorded or controlled, and ancillary evaluations were limited; future studies should prospectively capture ASM status, EEG findings, and standardized neuropsychological testing. Third, although manual segmentation is considered the gold standard for PVS quantification, we used the Potter visual rating scale, which is a semi-quantitative method and may not fully capture subtle changes in PVS burden. Additionally, diffusion-derived FW-WM may be sensitive to partial-volume effects, and increased PVS burden could bias a single global FW-WM estimate; future work should consider PVS-excluded masks or regional analyses. Fourth, while we performed laterality-aligned sensitivity analyses (ipsilateral/contralateral definitions relative to HS), we did not stratify all primary analyses by left- vs. right-sided HS as separate subgroups, which may leave residual heterogeneity related to seizure focus lateralization. Finally, sleep and circadian rhythm factors have a significant impact on GS function, but this variable was not controlled in the present study.

In summary, we studied GS function in patients with TLE-HS using multiple MRI-derived indices. A longer disease duration was associated with lower DTI-ALPS values, higher FW-WM levels, and larger CPV; moreover, smaller HPV related to both decreased DTI-ALPS and increased CPV. Taken together, these data indicate a statistical correlation between GS function and disease chronicity in TLE-HS and provide preliminary imaging evidence that may inform the development of GS-targeted therapeutic strategies.

## Data Availability

The raw data supporting the conclusions of this article will be made available by the authors, without undue reservation.

## Footnotes

1
https://www.nitrc.org/projects/cat/

2
https://www.fil.ion.ucl.ac.uk/spm/software/spm12/

3
http://surfer.nmr.mgh.harvard.edu/

4
www.fmrib.ox.ac.uk/fsl

5
https://markvcid.partners.org/
