# Supplementary material for: Glymphatic system dysfunction in temporal lobe epilepsy with hippocampal sclerosis: MRI-based evaluation of circulatory markers and disease progression
Source: Front Neurosci. 2026 Feb 19;20:1741257. doi: 10.3389/fnins.2026.1741257 (PMC12960557; doi:10.3389/fnins.2026.1741257)
Supplement: Supplementary file 1 [file Data_Sheet_1.docx]

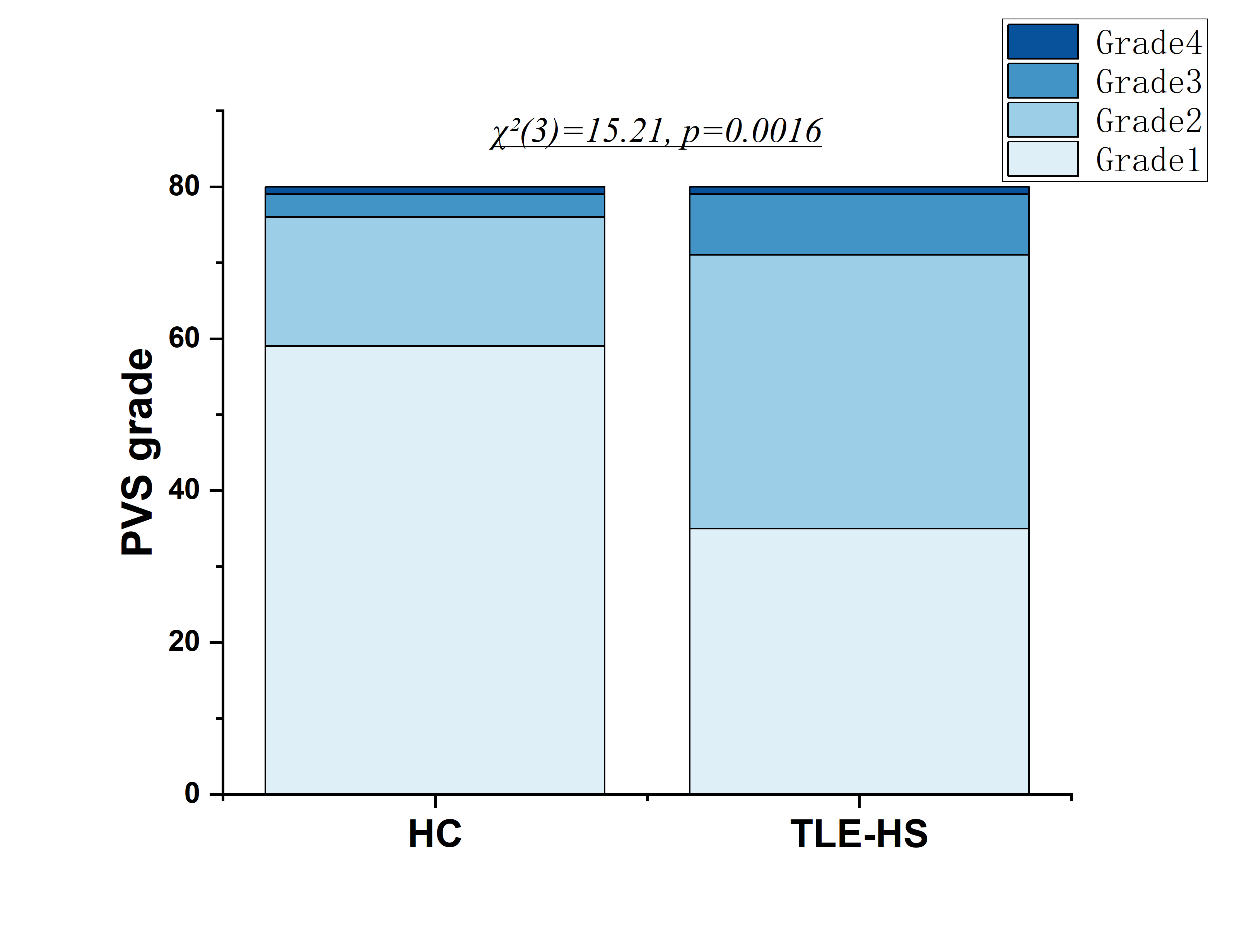


**Figure S1. Distribution of PVS-CSO grades in HCs and TLE-HS.** PVS in the centrum semiovale was rated using the Potter visual rating scale (grades 1–4). Group differences in grade distribution were assessed using Pearson’s chi-square test (χ² (3) = 15.21, p=0.0016; n=80 per group).

TLE-HS, temporal lobe epilepsy with hippocampal sclerosis; HC, healthy control; PVS-CSO, perivascular spaces in the centrum semiovale.
